# A prognostic model to identify short survival expectancy of medical oncology patients at the time of hospital discharge

**DOI:** 10.1016/j.esmoop.2022.100384

**Published:** 2022-02-07

**Authors:** M.A. Vicente Conesa, M. Zafra Poves, A. Carmona-Bayonas, I. Ballester Navarro, P. de la Morena Barrio, A. Ivars Rubio, S. Montenegro Luis, E. García Garre, V. Vicente, F. Ayala de la Peña

**Affiliations:** 1 Department of Hematology and Medical Oncology, Hospital Universitario Morales Meseguer, Murcia, Spain; 2 Department of Medicine, School of Medicine, University of Murcia, Murcia, Spain; 3 IMIB-Arrixaca, Murcia, Spain

**Keywords:** cancer, oncology, hospitalization, patient discharge, prognosis, nomogram

## Abstract

**Background:**

Hospitalization of cancer patients is associated with poor overall survival, but prognostic misclassification may lead to suboptimal therapeutic decisions and transitions of care. No model is currently available for stratifying the heterogeneous population of oncological patients after a hospital admission to a general Medical Oncology ward. We developed a multivariable prognostic model based on readily available and objective clinical data to estimate survival in oncological patients after hospital discharge.

**Methods:**

A multivariable model and nomogram for overall survival after hospital discharge was developed in a retrospective training cohort and prospectively validated in an independent set of adult patients with solid tumors and a first admission to a unit of medical oncology. Performance of the model was assessed by C-index and Kaplan–Meier survival curves stratified by risk categories.

**Results:**

From a population of 1089 patients with a first hospitalization, 757 patients were included in the training group [median survival, 43 weeks; 95% confidence interval (CI), 37-51 weeks] and 200 patients in the validation cohort (median survival, 44 weeks; 95% CI, 34 weeks-not reached). An accelerated failure time log-normal model was built, including five variables (primary tumor, stage, cause of admission, active treatment, and age). The C-index was 0.71 (95% CI, 0.69-0.73), with a good calibration, and adequate validation in the prospective cohort (C-index: 0.69; 95% CI, 0.65-0.74). Median survival in three predefined model-based risk groups was 10.7 weeks (high), 27.0 weeks (intermediate), and 3 years (low) in the training cohort, with comparable values in the validation cohort.

**Conclusions:**

In oncological patients, individualized predictions of survival after hospitalization were provided by a simple and validated model. Further evaluation of the model might determine whether its use improves shared decision making at discharge.

## HIghlights

• Hospitalization of poor prognosis oncology patients is a frequently missed opportunity for transition to palliative care.
• We developed and validated a prognostic index for cancer patients at hospital discharge based on five objective variables.
• Adequate prognostic stratification at discharge may facilitate transitions of care and shared decision making.

## Introduction

Admission to hospital is a negative prognostic factor for oncological patients. Regardless of the cause of admission, a median overall survival (OS) of ∼5 months is reported in some studies.^1^ Admissions related to symptom control are associated with even worse prognosis, with median survival usually <30 days,^2^ whereas hospitalizations generated by toxicity of chemotherapy usually portend a better prognosis. The poor life expectancy for patients after hospitalization is frequently not perceived by their physicians, however, and some publications point to a lack of correlation between the patient’s real prognosis and decision making, leading to inappropriate decisions and loss of end-of-life opportunities such as advanced directives or hospice referral.^3^^,^^4^ Since almost 60% of cancer patients are admitted to a hospital in their last 3 months of life,^5^ hospital discharge may well be a missed opportunity to guide decisions on palliative care planning. Providing physicians with better prognostic stratification tools at time of discharge might improve patient care after hospitalization and avoid overtreatment of patients with a limited life expectancy. No validated prognostic models, however, are available for oncological inpatients: tumor-specific prognostic scales are not easily applied to the characteristically heterogeneous population of cancer inpatients, whereas palliative prognostic scores are usually not applicable to patients under active treatment and only provide short-term prognosis estimations.^4^^,^^6^ An additional issue is that most palliative prognostic indexes include subjective evaluations such as clinical prediction of survival, which makes the use of these scales more difficult and less accurate, especially for less experienced physicians or for physicians who attend cancer patients only occasionally,^7^ for whom a more objective assessment of prognosis might be especially useful.

The purpose of this work was to develop and validate a prognostic index based on objective variables and potentially applicable to a broad, unselected population of medical oncology inpatients. The final aim of our work was improving care transitions, patient-centered treatment planning,^8^ and shared decision making for cancer patients at the time of hospital discharge.

## Methods

### Design

The study was designed in two phases: a first phase corresponding to the development of the prognostic model in a retrospective cohort and a second phase of prospective validation of the model. We evaluated a total of 1089 adult cancer patients with solid tumors (excluding lymphoma and hematological neoplasms). Only the first admission was considered for the model.

In the first phase, all consecutive patients with a confirmed solid neoplasm and with a first admission at the Department of Medical Oncology of Hospital Universitario Morales Meseguer, a Spanish public hospital, between January 2011 and May 2013, were retrospectively included. We initially included 957 patients; after excluding those patients with previous admissions (85 patients) or deceased in the hospital (115 patients), a final number of 757 patients formed the training cohort (Supplementary Figure S1, available at https://doi.org/10.1016/j.esmoop.2022.100384).

In the second phase of the study, 217 consecutive patients with a first admission to oncology and accepting the participation in the study were prospectively included during the first 72 h of hospital stay (December 2015 to March 2016). Patients deceased in the hospital (17 patients) were excluded, leaving 200 patients for the validation group. The study was approved by the Internal Review Board of the hospital (CEIC, code AVAL22/14), which waived written informed consent for the retrospective group. All patients included in the prospective set signed an informed consent.

Demographic and clinical data were retrieved from electronic medical records. Date of death was obtained from the national deceased registry (INDEF) when unavailable in the health records. For the development of the prognostic model, we only considered objective predictors, including demographic variables (sex, age, race), tumor-related data (site of primary tumor, stage at diagnosis, metastatic recurrence), treatment (active treatment with antineoplastic drugs in the last month or not), and admission-related variables (cause of admission, diagnosis at discharge, and length of stay). Tumor staging was based on *American Joint Committee on Cancer* (AJCC) 7th edition.

### Statistical analyses

The endpoint of the study was OS, estimated by the Kaplan–Meier method, and defined as survival from time of discharge to death by any cause (either cancer-related or not) or to last follow-up. Median follow-up was calculated by Kaplan–Meier analysis of censored patients.

Internal consistency, missing values, and outliers (for numerical variables) of each variable were revised before statistical analysis and extreme or discordant values were confirmed after reviewing the electronic health records. Since there were no missing data in the selected variables, no imputation techniques were used. Age was codified in three groups (<55 years, 55-80 years, >80 years) and the rest of the variables were grouped in a maximum of four categories. The selection of variables for the model was based on theoretical considerations, with three criteria: lack of dependence on the observer, the clinical relevance, and the direct availability of the data for the retrospective training dataset. To confirm that the selection of covariates was appropriate, a redundancy analysis was carried out to determine whether any of the covariates could be predicted from the rest of the variables. To further test the relevance of the variables chosen for the final model, we carried out both backward and forward stepwise selection based on minimization of Akaike inform criteria. The proportional hazards assumption for each covariate was checked using the Schoenfeld residuals test. Further insights on time-dependent effects were evaluated with Royston–Parmar flexible parametric survival models.^9^ This analysis was only exploratory and not included in the final model. To further confirm time-variant effects for each covariate, we used both a Cox model stratified by time intervals and resampling methods for fitting Cox models with time-varying coefficients. Potential interactions between covariates were also tested by adding a product term to the model and comparing it with the same model without interaction.

Since the data did not allow the assumption of hazards proportionality, a multivariable log-normal accelerated failure time model was built. A nomogram derived from the model was built to visually estimate the median survival and the probability of OS at 12 weeks, 24 weeks, and 1 year.

Model performance was assessed by calculation of Harrell’s concordance index (C-index), which considers right-censored data.^10^ The discrimination of the model was internally validated by bootstrapping (500 replications), generating an optimism-adjusted C-index for the model. The calibration at 1 year was analyzed with plots of observed (Kaplan–Meier survival estimate) versus predicted survival (model-derived probability of survival).

Additionally, we stratified patients into three prognostic groups, according to arbitrarily predetermined cut-offs for 24 week OS probabilities (<33%, 33%-66%, and >66%) as predicted by the model. Kaplan–Meier survival curves were obtained for each prognostic group and compared with the log-rank test. External validation of the model was carried out on the prospective cohort (*n* = 200), in which performance of the model was evaluated with the C-index and Kaplan–Meier curves in the same prognostic groups. A *P* value of 0.05 was set as statistically significant; all tests were two-sided. Statistical analyses were carried out with the *Hmisc*, *rms*, *survival*, *flexsurv*, *timereg*, *MASS*, and *rstpm2* packages from R software, version 3.6.1 (http://www.r-project.org). The TRIPOD Reporting Guidelines^11^ were followed for reporting the development and validation of the model.

## Results

### Patients and outcomes

The study population (757 patients included in the training group and the 200 patients included in the validation cohort) was selected from a population of 1089 patients with a first hospitalization in a Medical Oncology unit of a single Spanish public hospital. Patient characteristics are shown in Table 1.

**Table 1:** Characteristics of patients in the derivation and validation cohorts.

Clinical characteristic	Retrospective cohort *N* (%)	Prospective cohort *N* (%)	*P* value (χ^2^)
*N*	757	200	
Age (years), median (range)	64 (21-90)	61 (16-88)	0.46
Sex			0.28
Female	339 (45)	81 (41)	
Male	418 (55)	119 (59)	
Race/ethnicity			0.14
White	734 (97)	189 (95)	
Hispanic	14 (2)	3 (1)	
Other	9 (1)	8 (4)	
Primary tumor			0.08
Lung	176 (23)	56 (28)	
Gynecologic tumors	51 (7)	12 (6)	
Breast	156 (20)	30 (15)	
Head and neck	54 (7)	9 (4.5)	
Colorectal	105 (14)	30 (15)	
Urological neoplasms	43 (6)	18 (9)	
Digestive (non-colorectal)	98 (13)	20 (10)	
Pancreatic/hepatobiliary	47 (6)	14 (7)	
Other gastrointestinal tract tumors	51 (7)	6 (3)	
Other locations	74 (10)	25 (12.5)	
Stage			0.20
I-II	93 (13)	22 (11)	
III	153 (20)	31 (15.5)	
IV	511 (67)	147 (73.5)	
Cause of admission			0.006
Febrile neutropenia	139 (18)	18 (9)	
Other toxicities	47 (6)	13 (7)	
Other causes of admission	571 (76)	169 (84)	
Length of stay (days), median (range)	7 (1-119)	8 (1-43)	0.90
Active anticancer treatment	647 (85)	177 (88)	0.27
Referral to outpatient palliative program	210 (28)	62 (32.5)	0.18
Mortality (number of deaths) after discharge	554 (73)	150 (75)	0.60
Median overall survival, weeks (95% CI)	43 (37.4-51.4)	44 (33.8-not reached)	0.90

CI, confidence interval.

The comparison of the training and the validation sets did not show significant differences, except for the cause of admission. Stage IV or advanced disease was the predominant stage, and most patients (85%-88%) were on anticancer treatment. After a median follow-up of 41 months for the retrospective cohort [95% confidence interval (CI), 40.2-43.4 months] and 35 months (95% CI, 34.4-35.9 months) for the prospective cohort, 554 (73.2%) and 150 (75.0%) patients died, respectively. Median OS after discharge was similar for both cohorts: 43 weeks (95% CI: 37-51 weeks) in the training group, versus 44 weeks [95% CI: 34 weeks-not reached (NR)] among patients from the prospective validation cohort (Supplementary Figure S2, available at https://doi.org/10.1016/j.esmoop.2022.100384). Around 30% of patients in both groups were referred to outpatient palliative services at discharge.

### Development of the prognostic model and nomogram

After finding that the proportionality of hazards assumption was not fulfilled by a Cox model (global Schoenfeld residuals test, *P* < 0.001), we developed a multivariable log-normal accelerated failure time model. The prognostic model [Survival at Discharge of Oncological patients (SDO)] was fitted in the training group (*n* = 757). The final accelerated failure time log-normal multivariable model included five variables: stage, type of tumor (categorized as breast cancer, lung cancer, digestive non-colorectal, which also included hepatobiliary and pancreatic neoplasms, and other tumors), active antineoplastic treatment (yes or no), cause of admission, and age. No interaction or redundancy of variables was found and further testing with backward and forward stepwise selection of variables yielded the same set of covariates, except for age. Survival curves for each predictor are shown in Supplementary Figure S3, available at https://doi.org/10.1016/j.esmoop.2022.100384. An exploratory analysis with Royston–Parmar flexible parametric survival models^9^ showed the time-dependency of hazard ratios for stage, cause of admission, and active treatment. The high impact on survival of those three covariates was evident in the first 3 months after discharge, but decreased afterwards, thereby violating the proportional hazard ratios assumption. Additional analyses of these covariates were carried out with Cox model stratified by time intervals and with resampling methods for fitting Cox models with time-varying coefficients (data not shown), further confirming the time-varying effect of all covariates, except for age. Time-dependent hazard ratios for each covariate are shown in Supplementary Figure S4, available at https://doi.org/10.1016/j.esmoop.2022.100384.

The final model is defined by equation (1):

(1) Prob(T≥t)=1-φ{[log(t)-Xβ]/1.398385}, where

X β = 7.966781
  - 1.293278 (stage II)-1.745899 (stage III)-2.980672 (stage IV-advanced)
  - 0.5396663 (other tumors)-1.058724 (lung cancer)-0.8057411 (digestive non-CR tumors)
  - 0.8026972 (no active treatment)
  - 0.6127014 (admission: other toxicities)-0.8477568 (admission: other causes)
  - 0.2090313 (age 55-80)-0.4314865 (age >80 years) and c = (1) if subject is in group c, 0 otherwise

**Figure 1:**
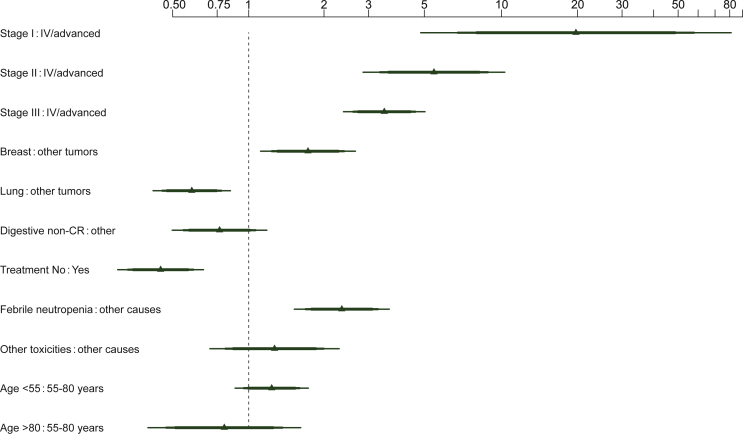
**Effect of survival at discharge of oncological patients (SDO) model predictors on overall survival after hospital discharge.** Estimated time ratios (TRs) correspond to the exponentiated coefficients of the multivariable log-normal accelerated failure time model; 90%, 95%, and 99% confidence intervals are provided. The most frequent category was taken as a reference for each variable. CR, colorectal.

**Figure 2:**
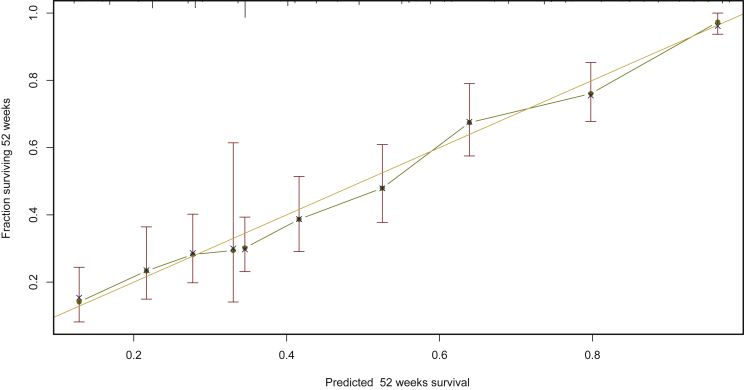
**Calibration plot of the model for the derivation group.** Calibration plot of the internal validation results in the derivation subset (*N* = 757) for 1-year overall survival.

In this log-normal parametric model, the exponentiated coefficients correspond to time ratios (TRs). The estimated TRs of the model are shown in Figure 1; a TR >1 is associated with longer survival and TR <1 with shorter survival. The model’s discrimination was fair, with a C-index of 0.71 (95% CI 0.69-0.73). After correction for optimism (internal validation with 500 bootstraps), the adjusted C-index was 0.70, with an *R*^2^, which corresponds to the proportion of the survival variability explained by the nomogram, of 0.35. The calibration of the model is shown in Figure 2, with excellent concordance between observed and predicted probabilities of 1-year survival.

**Figure 3:**
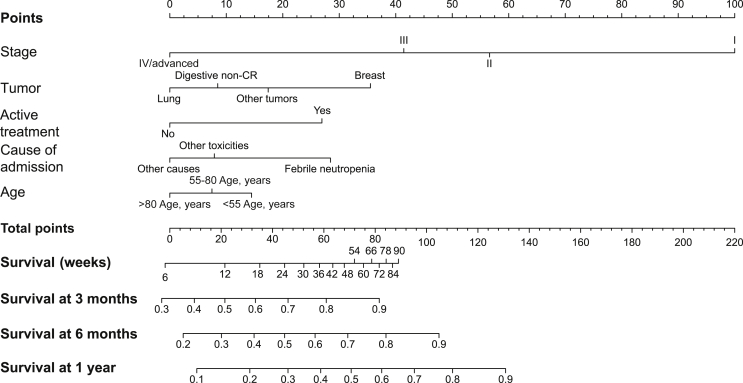
**Nomogram for the survival at discharge of oncological patients (SDO) model**. The points for each of the five variables are obtained by drawing a line upwards from the value of each variable to the points line. The sum of points for the five variables is marked in the total points line and a line perpendicularly drawn downward yields the expected survival and the probability of survival at 12 weeks, 24 weeks, and 1 year. CR, colorectal.

A nomogram derived from the SDO model allows a visual patient-specific estimation of median OS and of OS at 12 weeks, 24 weeks, and 1 year (Figure 3). The nomogram may be useful for easily identifying those patients with a short life expectancy after discharge. A Microsoft Excel-based calculator is also provided (Supplementary File S1, available at https://doi.org/10.1016/j.esmoop.2022.100384).

**Figure 4:**
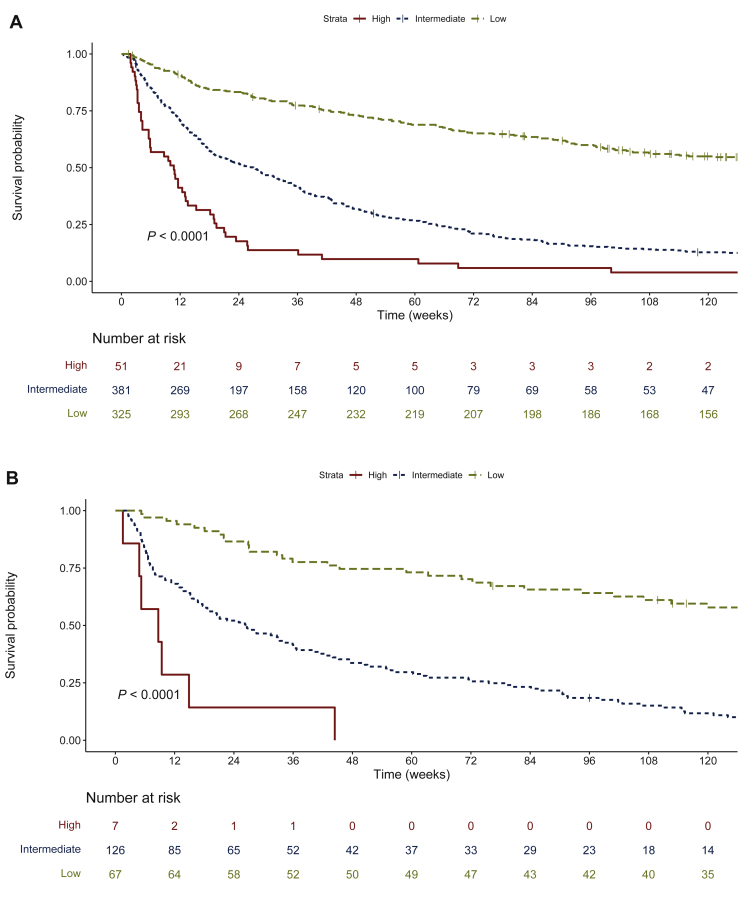
**Kaplan–Meier observed survival curves for prognostic groups in the training and validation cohorts.** Prognostic groups were defined according to predicted probability of survival at 24 weeks (high: <33%; intermediate: 33%-66%; low: >66%). (A) Training cohort (*N* = 757). (B) Prospective validation cohort (*N* = 200).

Although the model was intended to provide individualized survival predictions, we exemplified it by analyzing three arbitrarily predefined prognostic groups corresponding to <33% (*n* = 51), 33%-66% (*n* = 381), and >66% (*n* = 325) predicted probability of OS at 24 weeks, as calculated by the model nomogram. Median survival was 10.7 weeks (95% CI, 5.9-13.6 weeks), 27 weeks (95% CI, 20.1-32.3 weeks), and 156.7 weeks (95% CI, 116.0 weeks-NR), respectively (log-rank test, *P* < 0.001) (Figure 4A), thus demonstrating the model’s ability to prognostically stratify this heterogeneous group of patients.

### Prospective validation of the prognostic index

Validation of the model in the prospective cohort of patients discharged after hospitalization at the oncology unit (*n* = 200) showed a C-index of 0.69 (95% CI, 0.65-0.74), comparable to that obtained for the model in the derivation cohort. Kaplan–Meier curves for OS among the three predefined risk groups agreed with those obtained in the training group (Figure 4B), with median survivals of 8.7 weeks (95% CI, 4.8 weeks-NR), 29.4 weeks (95% CI, 17.8 weeks-NR), and not reached (95% CI, 112.6 weeks-NR) for the high-risk (*n* = 7), intermediate-risk (*n* = 126), and low-risk (*n* = 67) groups, respectively (log-rank test, *P* < 0.001).

## Discussion

The prognostic evaluation of oncological patients is necessary for therapeutic planning and shared decision making, but the heterogeneity of medical oncology patients admitted to a hospital converts prognosis in this setting in a complex challenge. We have developed a prognostic model (SDO) based on five accessible objective clinical variables that are significantly associated with OS. The prognostic model, available as a nomogram, agrees with quality criteria for prognostic scores, has been validated prospectively in an independent and more recent dataset, has a fair discrimination (C-index: 0.71), and seems easy to adopt in clinical practice.

The prognostic impact of unplanned hospital admission for cancer patients, with median OS <6 months for patients with any cause of admission and non-curable disease, has been shown before. In our series, the prognosis of patients admitted to the hospital was better, with a median OS ∼10 months, that corresponds to a highly diverse population of cancer patients, most of them under active anticancer treatment. This group of patients is less biased than other works focused on palliative cancer patients, thus improving the applicability of the model to a broader population of oncological inpatients. In this heterogeneous group, the SDO nomogram provided a good prognostic stratification at the time of discharge from the hospital.

The main advantage of the SDO model is its independence of the prognostic expertise of the clinician. Previous studies have demonstrated the prognostic value of performance status scales, such as Eastern Cooperative Oncology Group (ECOG), in the hospital or in outpatient settings. Beyond their lower precision, however, their administration is limited by subjectivity and variability among observers and by intra-individual variability during hospitalization,^12^, ^13^, ^14^ in contrast with their better discrimination in the outpatient setting.^15^ Other prognostic models, such as the palliative prognostic score (PaP score)^16^ or a palliative prognostic nomogram developed by Feliu et al.^17^ have obtained a similar discrimination (C-index between 0.70 and 0.73), but they are only useful for a predefined population of palliative care patients with advanced cancer, thus impending their application to the more heterogeneous population of cancer patients discharged from the hospital.^6^^,^^18^ Both scores also include a subjective evaluation of either the clinical prediction of survival by the physician, or the time since the diagnostic of terminal disease. Other models exclusively based on objective predictors, such as vital signs or laboratory data, are either restricted to patients receiving chemotherapy^19^ or are focused on advanced cancer patients previously identified as a palliative population.^20^ Finally, another recently published prognostic tool (IMPAC) for short-term mortality in hospitalized patients with advanced cancer is based only on objective data, although it is only applicable to one commercial electronic health record and the exact model has not been reported.^21^ In our model, the exclusive dependence of the prognostic index on a small number of objective clinical data might confer a higher robustness and applicability to the tool. In fact, although the training cohort was retrospective, the virtually equal performance of the SDO model in the prospective validation cohort is probably related to the model’s exclusive reliance on objective variables. Additionally, the direct availability of data on any electronic health record makes feasible its implementation as an automated clinical decision tool at the moment of discharge, or its easy calculation with a specific application, such as the Excel calculator here provided, or with a web-based calculator.

Considering the high rate of admissions of cancer patients to the hospital in the last months of life, admission is an opportunity to improve the continuity of cancer care. Some data, however, point to a lack of correlation between patient prognosis and decision making at the end of life for cancer patients. In our series, between 56% and 66% of the patients corresponded to high- or intermediate-risk groups for OS at 24 weeks, whereas only 30% of patients were referred to palliative programs. Although life expectancy is not the only factor for decisions related to palliative care,^22^ the introduction of prognostic models such as SDO may contribute to the individualization of patient trajectories, avoiding inadequate treatments^23^ and improving transition of care and advanced care planning for oncological patients.^24^^,^^25^ The introduction of similar opportunistic models, such as the initiation of palliative care in Emergency Departments, has shown an improvement in patients’ outcome in other settings.^26^ Similarly, those patients with a high or intermediate probability of death before 24 weeks at time of discharge from hospital, receiving or not active antineoplastic treatment, might be target groups for early and systematic integration of palliative care, an approach that has demonstrated increased survival^27^ and quality of life for patients with advanced cancer.^28^ Additionally, its integration with other clinical predictive models, such as those developed for assessing the risk of chemotherapy toxicity,^29^ might provide further refinements in the selection of patients for active treatment. For this specific goal of deciding on palliative chemotherapy, decisions relying only on performance status may not be associated with better results and considering also the life expectancy might likely result in avoiding treatments that do not improve the quality of life.^30^

Our work has limitations. First, although prospectively validated, the development of the model in the medical oncology unit of a single center and the lack of racial diversity of the sample may limit its generalizability. We consider that the diversity of tumor types and stages, however, together with a sufficient sample size make our results applicable to any general hospital in which cancer care is provided by multidisciplinary teams. The inclusion of predictive covariates which are not biased by a physician’s subjectivity also strengthens the applicability of the model in different settings, although multicentric testing of the model might be warranted. Second, it is possible that the integration of other clinical variables, especially the performance status, might further improve the model, although our aim was a model independent of any potentially subjective evaluation. An evaluation of the current model combined with ECOG should be carried out, however, preferentially after formal testing of interobserver concordance of ECOG assignment in the hospitalization setting. Third, the generation of a simple model based only on a few objective variables with all covariates categorized in two to four categories probably has been obtained at the cost of a loss of precision of the model, but we consider that a simpler tool might be easier to introduce in the daily clinical practice. A recent model for metastatic patients, that used high-dimensional data (4126 variables) from the electronic health record obtained a C-index of 0.74-0.78,^31^ but with a level of complexity that may limit its adaptation to other settings. Fourth, the SDO model is focused on evaluation at discharge, whereas other models have addressed the prognosis at time of admission. Since our goal was to improve the appropriateness of treatment along the continuum of care, however, and not only during the hospitalization, the stratification of patients at the time of discharge may provide a better assessment for outpatient palliative care integration. Finally, an alternative strategy might include an initial clinical evaluation to exclude patients with an obvious good prognosis or patients with far advanced tumors. Our first aim, however, was precisely to discriminate the different prognostic situations for oncological inpatients. The direct application of the SDO nomogram shows, in a pragmatic way, that a relevant group of patients who are actively treated during their admission to hospital and are not evidently identified as belonging to a poor prognosis group, actually have a limited life expectancy after discharge.

In conclusion, a simple and validated prognostic index (SDO) based on five observer-independent and easily accessible variables (age, type of tumor, cause of admission, tumor stage, and active treatment) can prognostically stratify oncological patients at discharge from hospital admission. Model-based identification of patients with a poor life expectancy after discharge might facilitate a better transition of care, avoiding the loss of palliative options for cancer patients. And providing patients with more accurate prognostic information should enhance their engagement in cancer treatment decisions, thus resulting in better patient-centered cancer care.
