# Supplementary material for: A prognostic model to identify short survival expectancy of medical oncology patients at the time of hospital discharge
Source: ESMO Open. 2022 Feb 7;7(1):100384. doi: 10.1016/j.esmoop.2022.100384 (PMC8844687; doi:10.1016/j.esmoop.2022.100384)
Supplement: Supplementary Figures S1-S4 [file mmc1.pdf]

SUPPLEMENTARY FIGURES

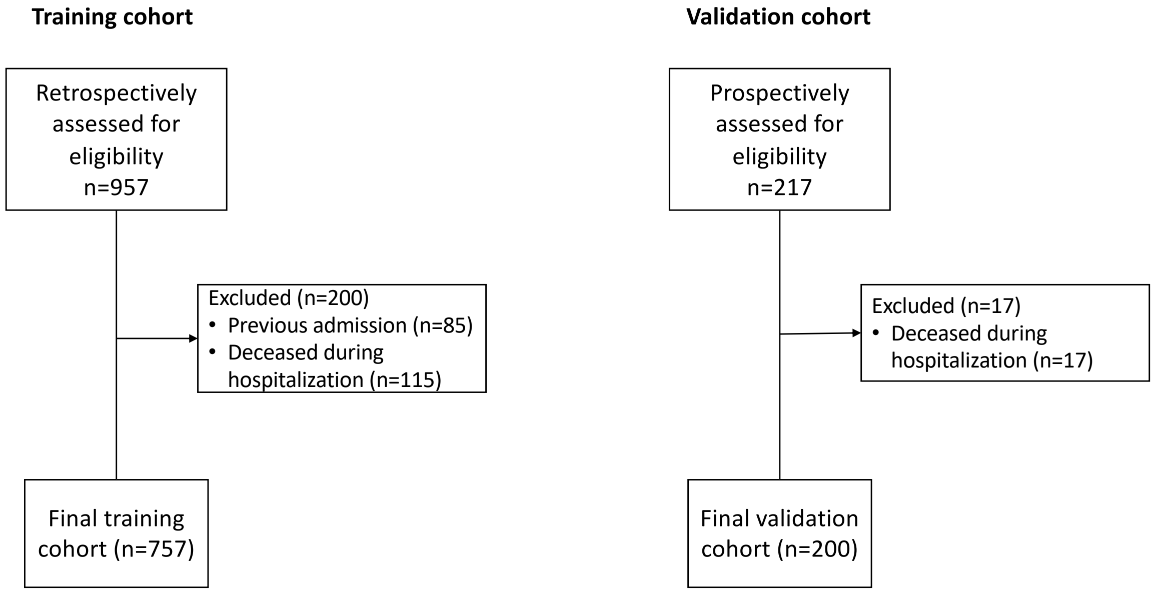

Supplementary Figure S1. Flowchart of patients included in the study. CONSORT flow diagram of training (retrospective and validation (prospective) cohorts.

A

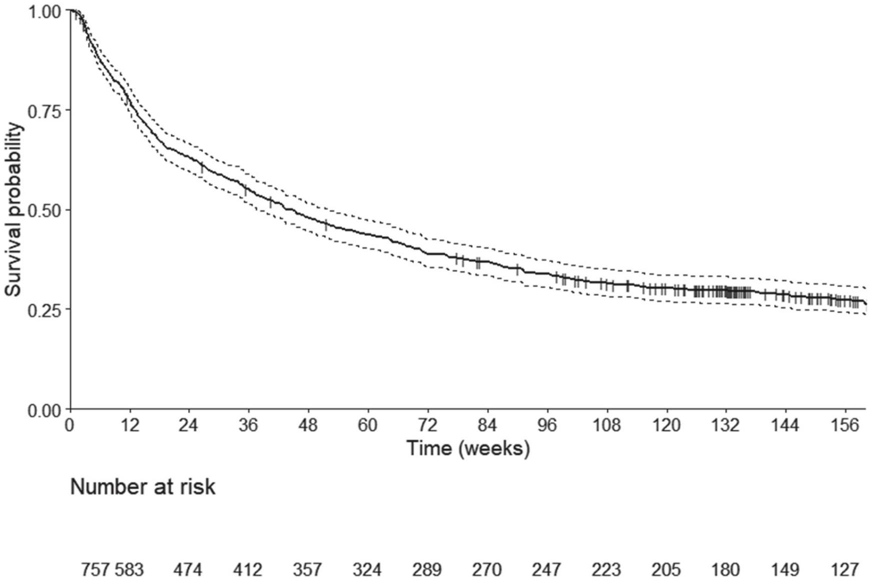

B

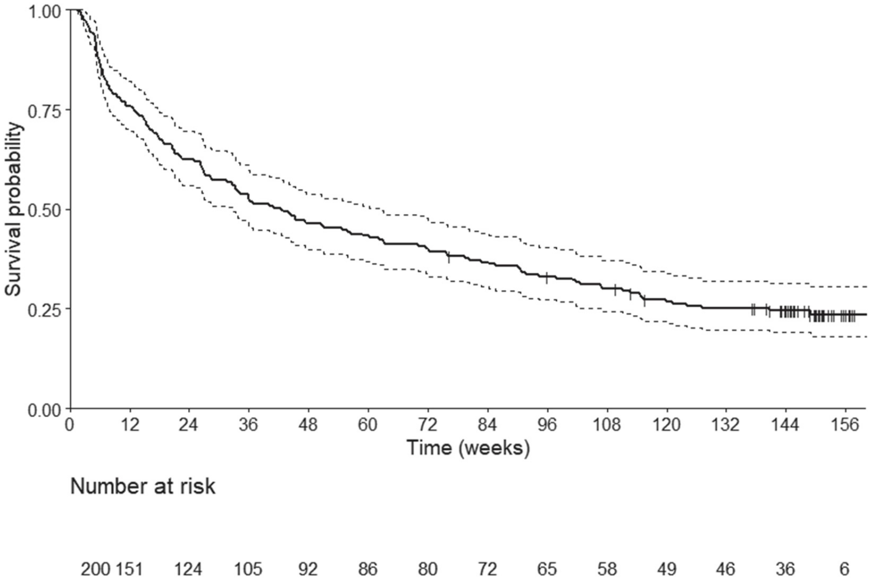

Supplementary Figure S2. Kaplan-Meier overall survival curves. A) Retrospective group (*N*=757). B) Prospective group (*N*=200). Overall survival estimation with 95% confidence interval.

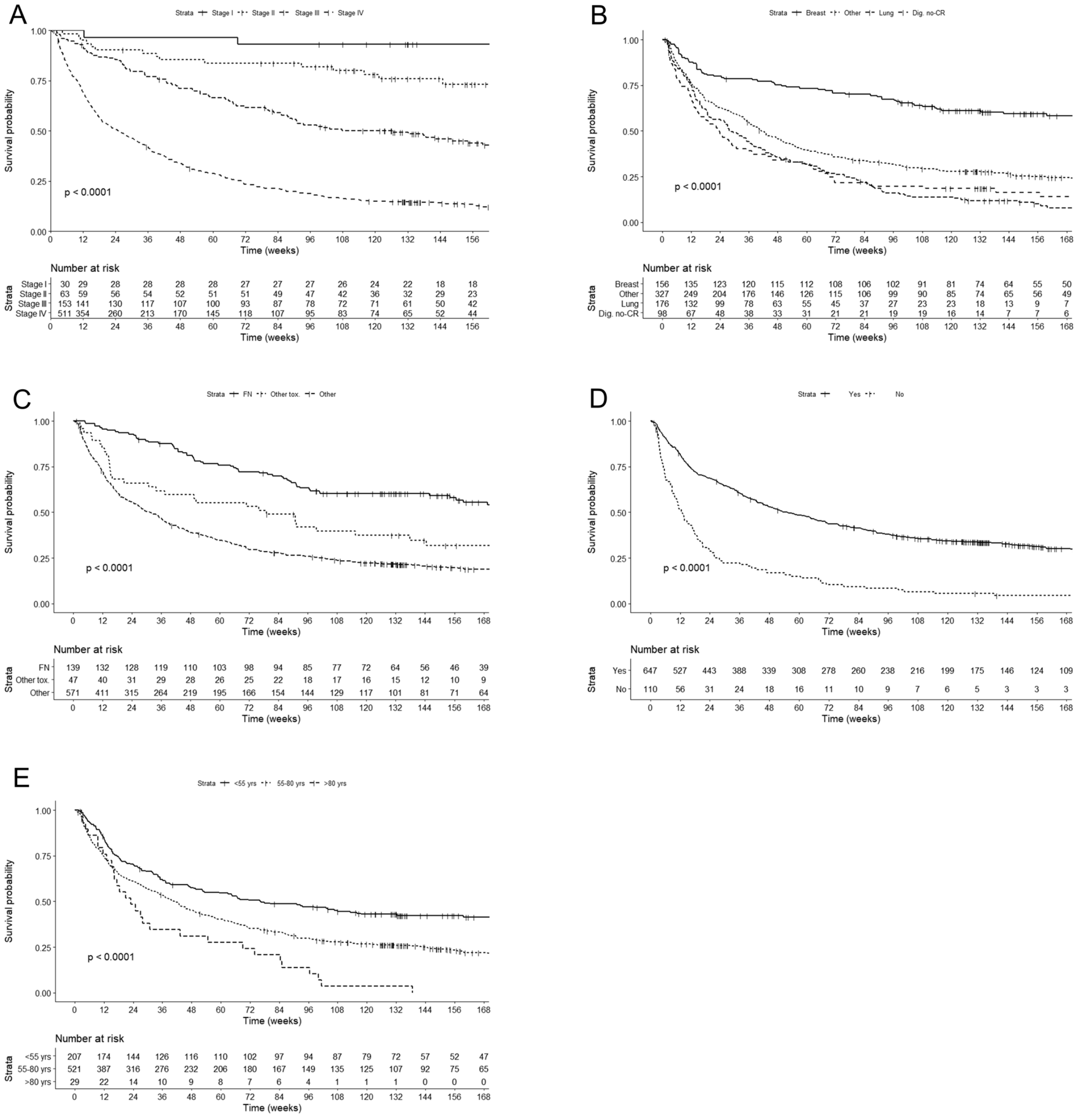

Supplementary Figure S3. Kaplan-Meier survival curves for predictors of the SDO multivariable model in the training cohort: A) Stage (I to IV), B) Type of tumor (breast cancer, lung cancer, other tumors, digestive non-colorectal), C) Cause of admission (febrile neutropenia, other toxicities, other causes of admission), D) Active treatment (yes/no), E) Age (<55 years, 55-80 years, >80 years). *P* values correspond to log-rank test for each variable.

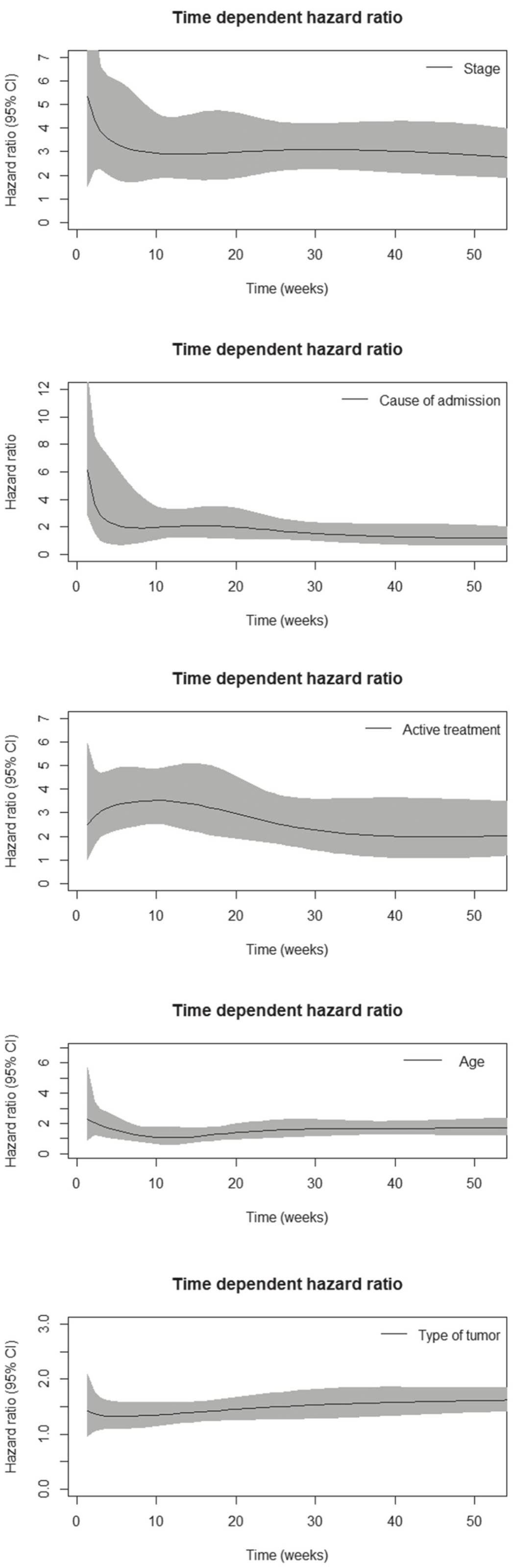

Supplementary Figure S4. Time-dependent hazard ratio curves for predictors of the SDO multivariable model in the training cohort. Time-dependent hazard ratio curves for each variable, corresponding to a univariate Royston-Parmar flexible survival model. This exploratory approach was not used for the final model and is hereby derived only to illustrate time-dependency of hazard ratios.
